# Isolation of cancer stem cells by selection for miR-302 expressing cells

**DOI:** 10.7717/peerj.6635

**Published:** 2019-03-26

**Authors:** Karim Rahimi, Annette C. Füchtbauer, Fardin Fathi, Seyed J. Mowla, Ernst-Martin Füchtbauer

**Affiliations:** 1 Department of Molecular Biology and Genetics, Aarhus University, Aarhus C, Denmark; 2 Cellular and Molecular Research Center, Research Institute for Health Development, Kurdistan University of Medical Sciences, Sanandaj, Iran; 3 Molecular Genetics Department, Faculty of Biological Sciences, Tarbiat Modares University, Tehran, Iran

**Keywords:** Stem cell marker, Cancer stem cells, MiR-302/367, Primary cell culture, G418 selection, Teratoma

## Abstract

**Background:**

Cancer stem cells are believed to be a major reason for long-term therapy failure because they are multi-drug resistant and able to rest mitotically inactive in the hypoxic center of tumors. Due to their variable number and their often low proliferation rate, cancer stem cells are difficult to purify in decent quantities and to grow in cell culture systems, where they are easily outcompeted by faster growing more ‘differentiated’, i.e., less stem cell-like tumor cells.

**Methods:**

Here we present a proof of principle study based on the idea to select cancer stem cells by means of the expression of a stem cell-specific gene. A selectable egfp-neo coding sequence was inserted in the last exon of the non-coding murine miR-302 host gene. As a stem cell specific regulatory element, 2.1 kb of the genomic region immediately upstream of the miR-302 host gene transcription start site was used. Stable transgenic CJ7 embryonic stem cells were used to induce teratomas.

**Results:**

After three weeks, tumors were removed for analysis and primary cultures were established. Stem cell-like cells were selected from these culture based on G418 selection. When the selection was removed, stem cell morphology and miR-302 expression were rapidly lost, indicating that it was not the original ES cells that had been isolated.

**Conclusions:**

We show the possibility to use drug resistance expressed from a regulatory sequence of a stem cell-specific marker, to isolate and propagate cancer stem cells that otherwise might be hidden in the majority of tumor cells.

## Introduction

Cancer is a diverse group of diseases and many aspects of carcinogenesis and tumor progression are still poorly understood. The stochastic and the cancer stem cell (CSC) models are two alternative explanations for carcinogenesis. In the stochastic model all transformed tumor cells have an equal tumor-forming potential, while the CSC model suggests that only CSCs, a small subpopulation of cancer cells, can generate new tumors (Holohan et al., 2013; Song et al., 2017).

CSCs were first described by Lapidot et al. (1994). They are defined as cancer cells that can self-renew and generate the heterogeneous lineages of a tumor (Clarke et al., 2006). This definition signaled a paradigm change because it linked tumor heterogeneity to multipotent CSCs and not to secondary mutations. CSCs are now recognized as one reason for therapy resistance and tumor relapse (Li et al., 2007). Like other stem cells, CSCs are multi-drug resistant (Wilson et al., 2011) and have a high level of DNA repair activity, which reduces their sensitivity to radiation (Baumann, Krause & Hill, 2008). CSCs are often slow growing and can rest in a quiescent state, which makes them insensitive to classical cancer therapies. The quiescent state bears the risk of tumor relapse from CSCs hiding as micro-metastases for long periods. The role of CSCs is partially obscured because CSCs are not a fixed self-renewing, but rather a dynamic population, which is in a kind of equilibrium with more differentiated tumor cells that might de-differentiate into CSCs (Gupta et al., 2011). It is still controversial whether CSCs are the only source of new tumors, but e.g., in colorectal cancer, CSCs were shown to be responsible for tumor progression (De Robertis et al., 2018). It is now generally recognized that CSCs are responsible for a large percentage of therapeutic failures (Batlle & Clevers, 2017). A deeper understanding of CSCs is required to develop better strategies to target these cells, however some of the stem cells properties hamper their analysis.

Most attempts to isolate or enrich CSCs utilize combinations of specific surface antigens (Medema, 2013), but transgenic approaches with reporter genes, driven by stem cell-specific promoters, have been used with tumor cell lines in culture (Bauderlique-Le Roy et al., 2015). Expression of SOX10/CD133 was used to isolate CSCs from adenoid cystic carcinoma (Panaccione et al., 2017). dTomato fluorescent protein driven by the promoter of *SOX2* led to isolation and characterization of human breast cancer cells (Liang et al., 2013). A similar strategy, with GFP driven by the *Arf* promoter, has been used to isolate perivascular cells from the primary vitreous of the mouse eye by FACS sorting (Iqbal et al., 2014). Such an approach should also be usable in experimental tumors in animals. However, because CSCs grow slower than the tumor cells they produce, it is still challenging to isolate and grow CSCs in culture.

Cell surface markers like CD133, CD24 and CD44 in colon cancer have been widely explored as stem cell markers because they are very suitable for FACS isolation of small stem cell populations (Sahlberg et al., 2014). Due to their functional relevance for “stemcellness”, stem cell specific transcription factors (TFs) like *OCT4*, *SOX2*, and *NANOG* have also been widely investigated (Luo et al., 2013). Like TFs, microRNAs (miRNAs) are involved in many cellular processes including “stemcellness” and cancer. Deregulation and the effect of miRNA expression pattern in liver and breast cancer stem cells have been investigated (Lou et al., 2018; Zhang, Xu & Zhang, 2018). Surprisingly, the use of miRNAs as markers for certain cell types has so far been little employed.

MiR-302/367 (here collectively called miR-302s) are a group of stem cell specific miRNAs. The miR-302 cluster is localized in the first intron of a non-coding host transcript. The primary host RNA includes three exons in human (Barroso-delJesus et al., 2008) and two exons in mouse (Rahimi et al., 2018a). MiR-302s alongside miR-200 have been reported as key regulators of stem cells behavior (Balzano et al., 2018). Furthermore, miR-302s have been shown to enhance the stemness of male germline stem cells (Zhu et al., 2018). Besides, expression of miR-302s is highly correlated with the expression of CSC markers (Volinia et al., 2014).

In human ES cells, expression of the miR-302 cluster is conferred by its immediate upstream regulatory region, located within 525 bp upstream of the transcription start site (Barroso-delJesus et al., 2008; Barroso-delJesus, Lucena-Aguilar & Menendez, 2009). In mice, we have shown that an extended regulatory sequence up to 2.1 kb, which is highly conserved between mice and humans, is involved in *miR-302* gene regulation (Rahimi et al., 2018a).

The aim of this proof of principle project was to utilize the expression of the stem cell-specific miR-302 host gene to isolate and select CSCs from a murine teratoma. This strategy utilizes the expression of the non-coding exons of the miR-302 host gene to express an egfp-neo fusion transcript. This reporter enables the selection of the CSCs expressing the miR-302 gene, by means of resistance to G418. Because expression of the egfp-neo is coupled to expression of a stem cell-specific gene, we speculated that CSCs can be kept in an undifferentiated stage until the G418 selection is relieved.

The specific advantage of the proposed strategy is that it can be used to isolate small numbers of slow growing cells because one can conditionally select for undifferentiated CSCs in culture.

## Materials and Methods

### Statistical data analysis

A two-tailed and unpaired t-test with Welch’s correction was used to compare the G418 selected and unselected cells on day three and four of differentiation separately. GraphPad Prism software version 7.00 for Windows was used to perform the statistical data analysis. Results are presented as mean ± standard deviation (SD). The value of ∗*p* < 0.05 was considered for statistically significantly level. The exact *p*-value is provided in the figure for each analysis.

### DNA and RNA preparation and PCR reactions

Genomic DNA was isolated according to Laird et al. (1991). Pfu DNA polymerase (Fermentas) or AmpliTaq Gold 360 Master Mix (ThermoFisher) were used for PCR and RT-PCR reactions. M-MLV Reverse Transcriptase (Invitrogen) kit was used for cDNA synthesis. Genomic positions refer to mouse genome version mm9.

### Cloning protocols

FastDigest enzymes (Thermo Scientific, Waltham, MA, USA) were used for all DNA restriction digestions. T4 DNA Ligase (Thermo Scientific EL0014) was used for ligation.

### The pmmiR302pGFP-NEO vector

The murine miR302pEGFP fragment was cut from the “pmmiR302pEGFP” vector (Rahimi et al., 2018b) with AseI and PaeI. This fragment included the murine miR-302 core promoter region from −595 to +45 (chr3:127,544,494-127,545,132) and the egfp CDS. The pUC19 (GenBank accession No. M77789.2) vector digested with NdeI and PaeI was used as a backbone to ligate the miR302pEGFP fragment in. The resulting vector “pUC19-pmmiR302pEGFP” was digested with XhoI and Bsp119I. The sequence coding for EGFP-NEO separated by the self splicing T2A peptide was obtained from the “pUC57-pPB.TET.GFP.RFP” vector (kindly provided by Dr. Mark Denham, Aarhus University) digested with XhoI and Bsp119I. The egfp-neo fusion sequence was ligated into the linearized “pUC19-pmmiR302pEGFP” to give the “pmmiR302pEGFP-NEO” vector (Fig. S1A). This vector was linearized with ApaLI for ES cell transfection.

### The pmmiR302hostGFP-NEO vector

In order to design a vector containing the whole murine miR-302 transcript including the egfp-neo sequence in the last (second) exon of the non-coding host RNA, the pKO Scrambler NTKV-1903 vector was used as the backbone. Using AscI, the neomycin gene cassette was removed and the vector was religated resulting in the pKO/noNEO vector. The DNA sequence of the egfp-neo fusion was obtained from the “pUC57-pPB.TET.GFP.RFP” vector by Bsp119I digestion and ligated into the dephosphorylated ClaI site of pKO/noNEO. The first part of the gene cassette excluding the promoter, was amplified by Pfu polymerase using fwd: 5′ AAGAATACCGGTAGAACAGGACTCTTTGGG; rev: 5′ AAGAATGGATCCGGGATTTGCCTTTGTGGA primers which included AgeI and BamHI sites respectively. The length of this sequence is 1,603 bp (chr3:127,545,106-127,546,708) starting from the transcription start site, spanning the intron and ending 76 bp in the second exon. The PCR product was ligated into the AgeI and BamHI sites of the vector.

The second part of the gene cassette was amplified using Pfu polymerase with the following primers fwd: 5′ AAGAATCCGCGGCAATCCAGCTATGAGTAACA; rev: 5′ AAGAATGTCGACAGAAGGGATGAGGGAGAG, which include SacII and SalI sites respectively. The length of this sequence is 1,756 bp (chr3:127,546,782-127,548,537), starting 148 bp downstream of the second exon. The PCR product was cloned into the SacII (not FastDigest) and SalI sites of the vector. This resulted in the “pmmiR302hostGFP-NEO” vector (Fig. S1B).

### PGKmmiR302hostGFP-NEO

In order to investigate the processing of the miR-302 host-egfp-neo fusion RNA, the PGK promoter was inserted upstream of the mmiR-302 host gene transcription start site. The PGK promoter was cut from a vector with HindIII and PstI and inserted in the pUC18 vector (GenBank: A02710.1) which was digested with the same enzymes. This new vector was digested with PstI and SalI and ligated to a PstI and SalI digested PCR product, which had been amplified from the “pmmiR302hostGFP-NEO” vector using fwd: 5′ AAGAATCTGCAGAACTCAGGAGTTAGGAGTAG; and rev: 5′ AAGAATGTCGACCATGTTAAAGCAGAGGGGA primers. By digesting the “pmmiR302hostGFP-NEO” vector with NheI and SmaI, 3 different fragments were achieved. The 3,757 bp fragment (including miR-302 host transcript + egfp-neo) was ligated to the backbone of the new “pUC18-PGKp” vector after digestion with NheI and SmaI. The obtained vector “PGKmm302hostGFP-NEO” (Fig. S1C) was linearized with SspI and used for the ES cell transfections.

### The pmmiR302phostGFP-NEO vector

A 2,428 bp PCR amplicon including 2,120 bp of the miR-302 regulatory sequence (chr3:127,542,969-127,545,378) was amplified using forward primer 5′ AAGAATACCGGTCTGGAGTTGCTTTGTTTTC including an AgeI site and reverse primer 5′ AAGAATCATGTTAAAGCAGAGGGGA including a SpeI site. Digestion of the 2,428 bp PCR product with AgeI and SpeI gave two fragments of 2,249 and 176 bp respectively. By digestion the vector “pmmiR302hostGFP-NEO” with AgeI and SpeI, a fragment of 112 bp was removed and replaced with the 2,249 bp of the digested PCR product. This new vector, “pmmiR302phostGFP-NEO” (Fig. S1D), was linearized by AseI and used for CJ7 cell transfection.

### Cell culture and electroporation

CJ7 murine ES cells (Swiatek & Gridley, 1993) were grown in ES cell medium [DMEM (Gibco 41965-039), 15% Fetal Calf Serum (2602-P250915; Pan Biotech GmbH, Aidenbach, Germany), 1000 U/ml LIF (Invitrogen PMC9484; Invitrogen, Carlsbad, CA, USA), 1% glutamine (Gibco 25030; Gibco, Waltham, MA, USA), 1% Penicillin-Streptomycin (Gibco 15070-063), 1% non-essential amino acids (Gibco 11140), 1% fresh 10 mM β-mercaptoethanol, 1% 100X nucleosides mix] on mitotically inactivated feeder cells, unless otherwise mentioned. All cell culture dishes were gelatinized with 0.1% gelatin (Sigma-Aldrich G1393; Sigma-Aldrich, St. Louis, MO, USA). Electroporation settings were 240 V and 500 µF, 6.6*10^6^ cells in 800 µl complete PBS. Electroporated cells was washed in 10 ml ES cell medium and seeded on 4*6 cm dishes, which were pre-seeded with feeder cells. For all transfections, 25 µg vector were linearized and ethanol precipitated. Selection of the transfected cells was started 24–36 h after electroporation. Colonies were picked after 6–8 days selection with 350 µg/ml G418 at a potency of 785 µg/mg (04727894001; Roche Diagnostics GmbH, Basel, Switzerland).

### Teratoma derived cells

Generation of teratomas and isolation of cells is described in (Rahimi et al., 2018a). Briefly, for each injection, 1,600 stably transfected CJ7 ES cells with “pmmiR302phostGFP-NEO” vector suspended in 50 µl Hank’s buffer were used. The cells were injected subcutaneously and bilaterally into the back of two seven-month-old 129Sv/Pas isogenic male mice. After 21 days, when tumors had reached a size of around 1 cm^3^, mice were sacrificed by cervical dislocation and tumors harvested carefully. For primary cell culture, tumors were cut in 3–4 mm^3^ pieces, washed three times with cold calcium and magnesium-free PBS, immersed in 0.25% trypsin for 6 h at 4 degrees and, after removing excess trypsin, incubated at 37 degrees for 30 min. Trypsin was inactivated by FBS and, 10^6^ cells were seeded per well of a 12 well plate in ES cell medium or feeder cell medium with and without G418 (350 µg/ml). Finally, teratoma derived ES cell-like cells were used for further analysis.

### Microscopic analysis

All fluorescence images were taken with a Leica DMIL microscope using a Leica DFC 425C camera. Bright field images were taken with an inverted Leica DMIL microscope with Hoffmann contrast.

### Ethics Statement

Animal experiments were done according to the regulations of the Danish Animal Experiments Inspectorate, the legal authority under the Danish Ministry of Environment and Food (permission number: 2015-15-0201-00517).

## Results

### Egfp-neo expression driven by murine miR-302 proximal promoter in transgenic CJ7 murine ESCs

In order to select transfected ES cells expressing the murine miR-302 host gene, an egfp-neo fusion cassette was made in which the egfp-neo was driven by the miR-302 core promoter (Fig. S1A). This predicted miR-302 upstream regulatory sequence included the region from −595 to 45 bp of the first exon. CJ7 ESCs were electroporated with a linearized construct to promote random integration. About 80% of the G418 selected colonies showed a weak but clear EGFP signal. To confirm that the expression of the miR-302 reporter is stem cell specific, we grew the selected clones in differentiation medium without LIF and feeder cells. Under such conditions, the EGFP signal was almost lost within five days (Fig. S2).

To further analyzing EGFP expression from the miR-302 promoter, 228 primary colonies were picked and grown under ES cell condition with LIF and feeder cells as well as in ’differentiation’ medium. Of these 228 clones, 118 survived under ES cell condition and 100 under differentiation condition. After five days under these culture conditions, 93% of colonies in ES cell medium showed partial to complete EGFP expression compared to 35% of colonies in differentiation medium (Table S1), confirming that expression of the mmiR-302 reporter depends on the differentiation status of the cells.

In a few clones, significant EGFP expression was found after five days under differentiation conditions (Fig. S3). While this could be due to enhancer activity flanking the integration site, the microscopic image of the green colonies indicated a reappearing of undifferentiated cells as is frequently seen in ES cell cultures under these conditions.

### Murine miR-302 host RNA can support coding sequence

Murine miR-302 host RNA is a non-coding transcript of unknown function. The RNA is spliced, poly-adenylated and exported from the nucleus (Rahimi et al., 2018a) and thus might be used to introduce a coding sequence to be translated. In order to test this, the fused coding sequences of egfp-neo were introduced into the second exon of the miR-302 host RNA under the control of the PGK promoter (Fig. S1C). Transfected CJ7 ES cells were selected using G418. Twelve randomly selected clones were analyzed by RT-PCR (Fig. S4) and fluorescent microscopy (Fig. S5). RT-PCR results showed expression and correct splicing of the egfp-neo containing murine miR-302 host transgene.

### Egfp-neo expression driven by the extended murine miR-302 promoter

Having established that the miR-302 host gene can support translation of a reporter gene, we replaced the PGK promoter with 2,120 base pairs of upstream genomic region (Fig. S1D), which we had found to contain additional promoter/regulatory sequence responsible for miR-302 host gene expression (Rahimi et al., 2018a). Murine ES cells were transfected with this construct and selected with G418. The number of clones obtained was significantly less compared to what had been observed using the PGK promoter, confirming, that the miR-302 promoter is weaker than the PGK promoter. Eighteen resistant clones were analyzed and contained the complete transgene cassette.

**Figure 1:**
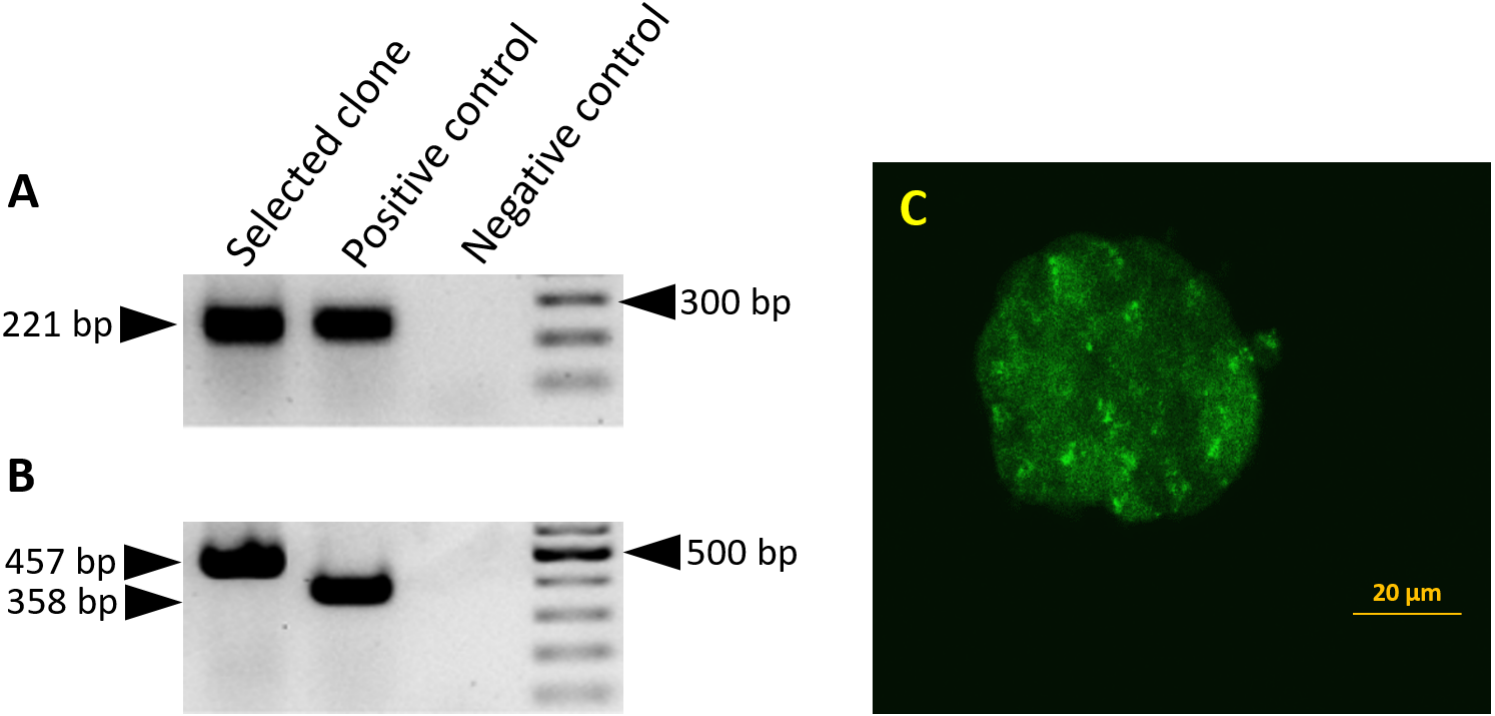
Splicing of the miR-302 host transgene. Detection of *miR-302* splicing in transgenic ES cells harboring the 2.1 kb upstream genomic region and the miR-302 host gene with egfp-neo in the second exon. Primers bind in exon1 and 2, the unspliced RNA would give a PCR product which is 1,487 bp larger. (A) The spliced endogenous *miR-302* transcript results in a 221 bp band. (B) After splicing, transgenic *mmiR302-egfp* transcript results in a 457 bp band. The positive control is cDNA from a transgenic teratoma in which exon 1 is fused to egfp with an expected band size of 221 bp for the endogenous *miR-302* in A and 358 bp for *miR302-egfp* in B*.* Negative control is the PCR reaction without cDNA template. (C) Transgenic ES cells showing a weak but distinct fluorescence signal. Scale bar is 20 µm. Cells from this clone were used to generate teratomas.

Correct splicing of the miR-302-egfp-neo transcript was confirmed by RT-PCR in all eighteen transgenic clones. EGFP expression was confirmed by fluorescent microscopy in the transgenic clones, even though the EGFP expression level was weaker compare to the EGFP signal driven by PGK promoter. One of these clones was selected for the generation of teratomas (Fig. 1).

### Primary cell culture of teratoma derived cells

In order to test our hypothesis that cancer stem cells can be selected for by the miR-302 gene driven expression of neo resistance, we generated teratomas by subcutaneous injection of transgenic ES cells into isogenic mice. Three weeks after injection, when the teratomas had reached a size of around 1 cm^3^, the mice were sacrificed and the tumors harvested and divided for primary cell culture and RNA isolation for molecular analysis.

**Figure 2:**
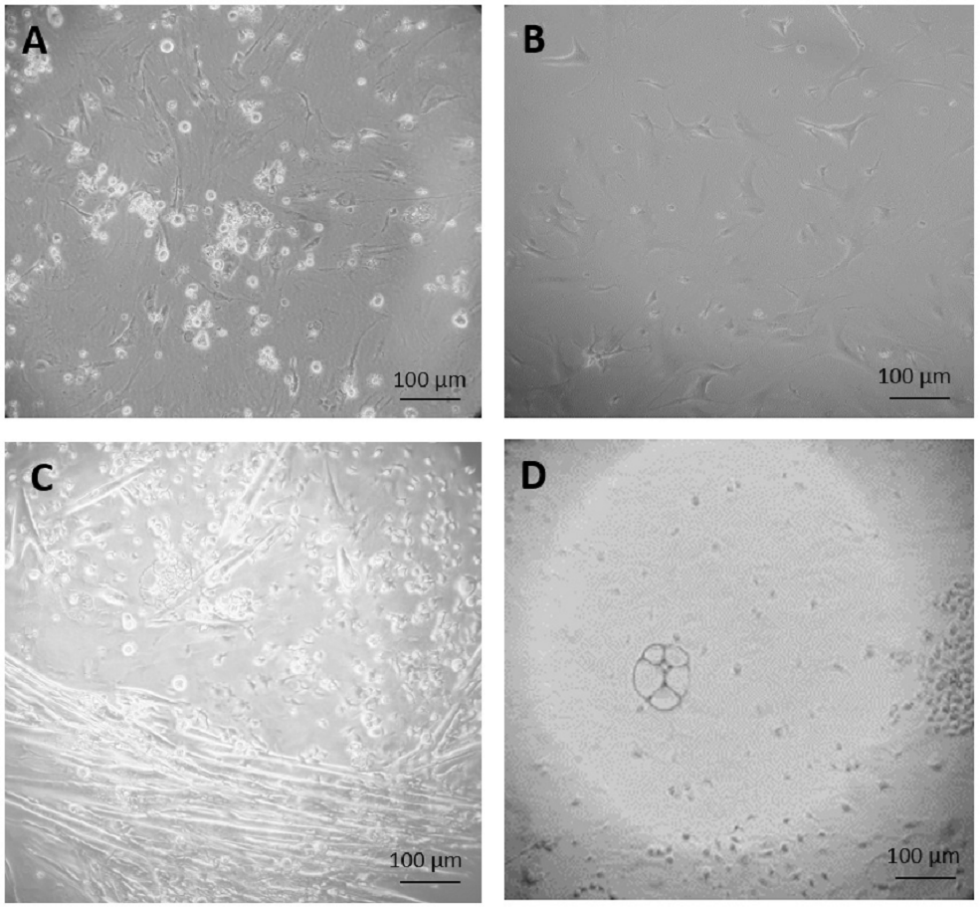
Primary culture of cells derived from teratoma. (A) After three weeks of culture, none of the cells grown on feeder cells in ES cell medium survived selection by 350 µg/ml G418. (B) Fibroblast-like cells, after 34 days culture in feeder medium without G418. (C and D) After two weeks culture in feeder free culture in ES cell medium without G418, some early myotubes, possibly adipocytes, and some fibroblast-like cells appeared (C). In addition, many small, rapidly growing cells appeared which were not very adhesive (D). Cells are visualized by Hoffmann contrast and the scale bar is 100 µm.

The tumors were treated with trypsin in order to isolate single cells, which were cultured under the following four conditions: ES cell medium with and without G418 and feeder cell medium with and without G418. Cells under G418 selection were cultured on mitotically inactivated feeder cells, cells in medium without G418 were grown feeder free. Neither in ES cell nor in feeder medium, the primary cells survived G418 selection, indicating a low number of stem cells (Fig. 2A). After three weeks of culture in feeder cell medium without feeder cells and without selection, some fibroblast like mesenchymal cells appeared (Fig. 2B).

After two weeks of culture in ES cell medium on gelatinized dishes without feeder cells and without G418 selection, some fibroblast-like cells, some differentiated early myotubes and possible adipocytes appeared (Fig. 2C). We speculated that this culture might contain miR-302 positive stem cells that we had missed in the culture, which was under immediate selection due to their low number. In order to test this, the partially differentiated culture shown in Fig. 2C, was split in two, one part was grown in ES cell medium and one in feeder medium, both containing G418. In this way, it was possible to distinguish if the miR-302 promoter activity was dependent on the medium condition and thus bona fide on the differentiation status of the cells.

**Figure 3:**
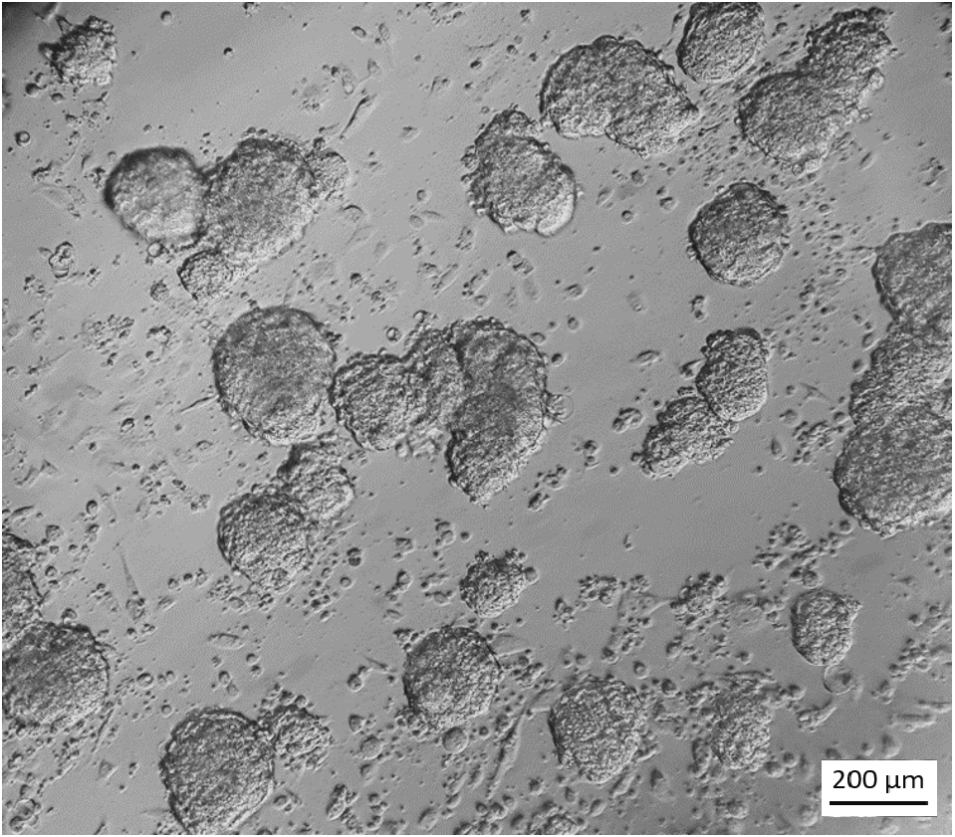
Appearance of stem cell like cells under selection. 18 days after starting G418 (350 µg/ml) selection on secondary cultures grown in ES cell medium, colonies with an ES-cell-like morphology were visible while other cells had died. Cells are visualized by Hoffmann contrast. The scale bar is 200 µm.

In feeder medium with G418, no cells survived, indicating that cells which can grow in feeder medium do not express NEO under the control of the miR-302 regulatory region. In contrast, if the same cells were grown in ES cell medium with G418, stem cell like colonies started to appear after two weeks while the differentiated cells died (Fig. 3).

**Figure 4:**
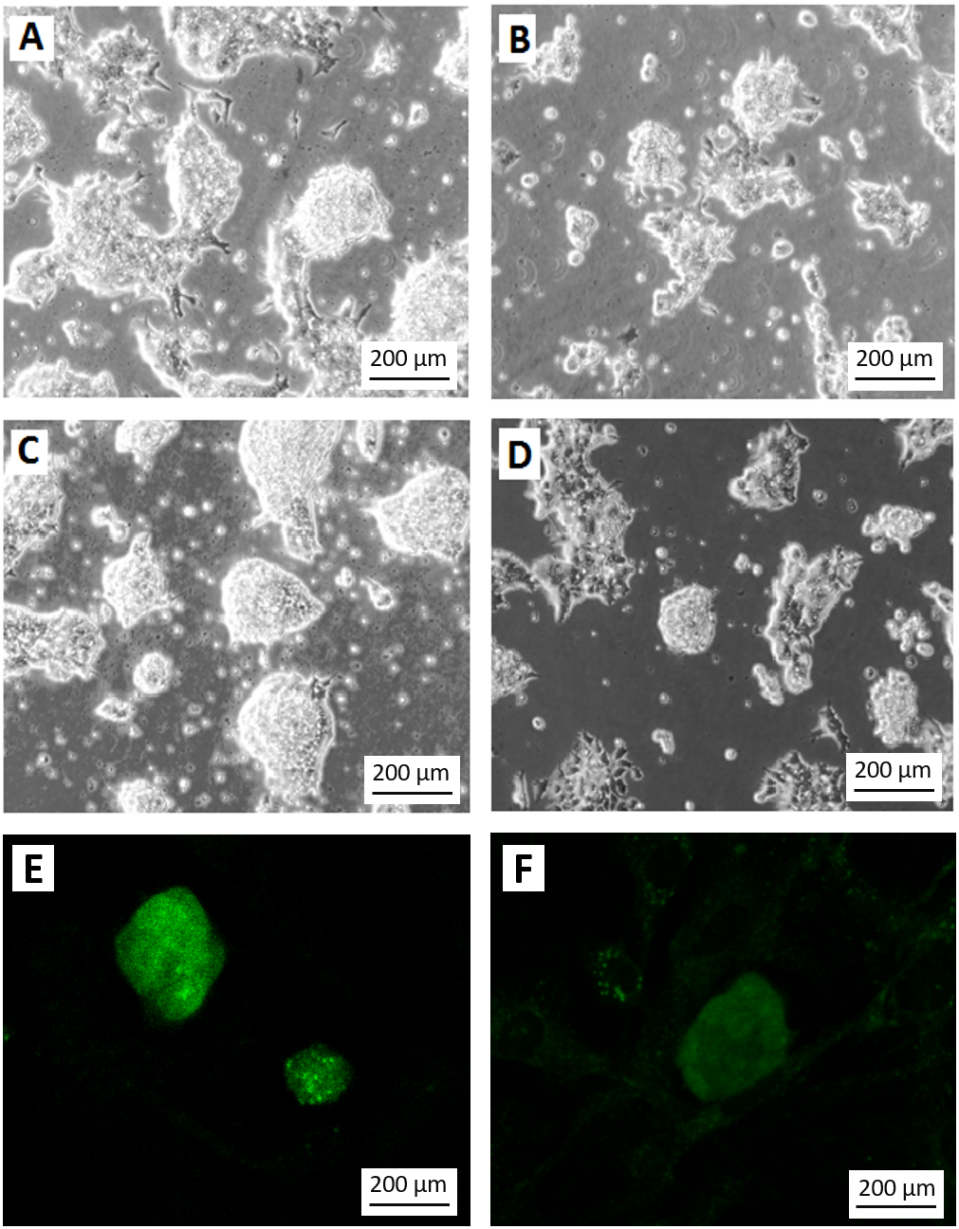
Differentiation of teratoma derived cancer stem cells without G418 selection. Morphology of the stem cell-like colonies grown in ES cell medium with (A, C) and without (B, D) G418 (350 µg/ml) for three (A, B) and four (C, D) days. Without selection, the cells rapidly lost their stem cell morphology. Note the disappearance of smooth slightly asymmetric colonies in which individual cells are difficult to recognize. Without selection, only very few colonies appeared undifferentiated, like the one in the center of (D). (E) EGFP expression signal from colonies grown under the same condition as in (C). (F) Weak EGFP signal in colonies grown under the same condition as in (D). The weak fluorescence is most likely due to residual EGFP protein. Cells in A to D are visualized by Hoffmann contrast. The scale bar is 200 µm.

To test if the selection for the NEO resistance coupled to the miR-302 expression is necessary to keep the population of growing cells undifferentiated, we compared the colony morphology of cells grown for three or four days after passaging either with or without G418. Without selection, the morphology of the colonies changed dramatically even though the culture conditions were otherwise identical (Figs. 4A–4D). Furthermore, colonies under selection expressed visible amounts of EGFP (Fig. 4E), while the fluorescence signal became very weak within 4 days without selection (Fig. 4F). The weak signal is most likely due to the half life time of the EGFP protein but might also be found in individual less differentiated colonies as the one mentioned in Fig. 4D.

**Figure 5:**
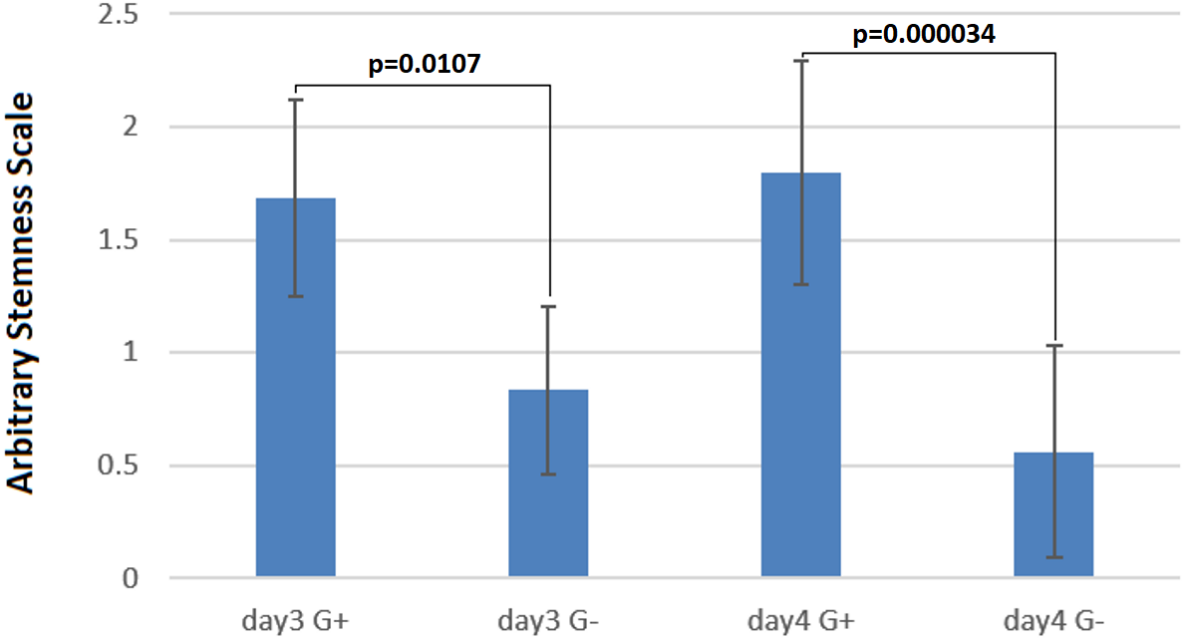
Cells under G418 selection are prevented from differentiation. On the arbitrary “stemness” scale, a completely differentiated culture would have the value 0 while a culture with all colonies undifferentiated would have a value of 2.5. The difference between cells with and without G418 is statistically significant after three days, but becomes more pronounced after four days. Data are shown as mean ± SD, and *p < 0.05 as significantly level. Two-tailed and unpaired *t*-test with Welch’s correction was performed for comparing G418 selected and unselected samples in days three and four of differentiation separately.

To quantify the stemness of these cells, two independent experienced persons, who did not know the culture conditions, scored colonies on 29 micrographs randomly taken from the four above-mentioned cultures (Figs. 4A–4D). This scoring showed clearly, that the cultures without selection were more differentiated (Fig. 5) than those under G418 selection. This confirmed our hypothesis that it is possible to derive and culture stem cells from tumors by coupling neo resistant expression to the expression of a stem cell marker like mmiR-302.

In order to detect the spliced egfp-neo RNA in the ES cell like teratoma derived cells with and without G418 selection for four days, we performed RT-PCR from exon 1 to exon 2 spanning the egfp-neo. This showed that the initially weak transgene expression was upregulated in cells stably grown under selection (compare lane 1 and 2 in Fig. S6), but that the cells started to differentiate and down regulate miR-302 expression when selection was released (compare lane 2 and 3 in Fig. S6). The short amplicon of the endogenous miR-302 host RNA (221 bp) is under these conditions amplified beyond the exponential phase and does therefore not reflect the differences between the culture conditions.

## Discussion

CSCs are an essential factor in cancer relapse (Teng et al., 2018) and an obvious target for cancer treatment (Cianciosi et al., 2018). However, our knowledge about CSCs is limited due to the inherited difficulty to obtain pure populations of these cells. In this proof of principle study, we show that it is possible to isolate CSCs from tumors by expressing a resistance gene under the control of a stem cell-specific regulatory element. Here, the miR-302 host gene was used, including 2.1 kb of the upstream genomic region and a neomycin resistance gene introduced into the second exon. We have shown that the non-coding miR-302 host RNA is capped, spliced and exported from the nucleus (Rahimi et al., 2018a), and thus speculated that it might be possible to turn it into a coding mRNA. It is an interesting side aspect of our work, that this transgenic hybrid RNA can support the translation of a coding sequence. We used ES cell-derived teratomas as a well-established model of tumors (Tzukerman et al., 2003). It could therefore be argued, that the stem cells we isolated were residual ES cells rather than CSCs. Two observations strongly indicate that this is not the case. The CSCs obtained after selection grew faster than CJ7 ES cells and, importantly, without selection the cells started to differentiate under culture conditions in which ES cells will proliferate without differentiation (Figs. 4B and 4D). In an other study, we have tested the expression of a luciferase reporter gene under the control of the same 2.1 kb genomic region. Also in that assay, expression was lost when the selection pressure was released, further indicating that the CSCs are different from ES cells (Rahimi et al., 2018a). Our results confirm that the 5′ upstream genomic region of the miR-302 host gene can drive the expression of a reporter gene in a stem cell-specific pattern. These results are in agreement with the report of Barroso-delJesus, Lucena-Aguilar & Menendez (2009) showing that human miR-302 is involved in the maintenance of stem-ness and self-renewal of human ES cells, an effect that is mediated by the regulation of cyclin D1 and D2 expression, that affect the balancing of the cell cycle (Card et al., 2008; Lee et al., 2008).

It was surprising that no cells survived the G418 selection in the initial culture, even though we know that it must have contained stem cells. This is evident because we could select plenty of stem cells from the differentiated culture which was prepared by the same pool of cells. There are several reasons why the original selection might have failed. The primary cells from the tumor might be specifically stressed by the abrupt switch from a relatively hypoxic tumor environment to a cell culture environment and thus might be especially sensitive to the selection. Likewise, might conditions like cell density etc. make the cells more vulnerable? We can therefore not exclude, that different types of tumors need different types of primary culture conditions. We are also aware of the fact that CSCs from teratomas are particularly fast growing, which might have eased the selection in our example. However, the fact that the stem cells were hidden in the differentiated culture consisting of a large number of bona fide post mitotic cells, shows that the CSCs were by no means overgrowing or outcompeting more differentiated cells in the culture.

## Conclusions

In order to isolate cancer stem cells from primary tumors, we performed a proof of principle study in which we selected for cancer stem cells expressing the neomycin resistance gene under the control of the stem cells specific miR-302 host gene promoter. In this transgene, the resistance gene was inserted into the last exon of the long non-coding miR-302 host gene, which to our knowledge is a new strategy. Our result confirm that it is possible to isolate cancer stem cells by means of their stem cell specific miR-302 expression and that it is possible to maintain the stemcellness of these cells by continued selection.

##  Supplemental Information

10.7717/peerj.6635/supp-1 Supplemental Information 1 Isolation of cancer stem cells by selection for miR-302 expressing cells

10.7717/peerj.6635/supp-2 Supplemental Information 2 Original Gel Pictures

10.7717/peerj.6635/supp-3 Data S1 Stemness state scoring of the teratoma derived cells with & without G418 Two independent and stem cells experienced persons scored the morphology of 29 micrographs taken from teratoma derived cells growing with and without G418 for 3 and 4 days.

## Additional Information and Declarations

### Data Availability

The following information was supplied regarding data availability: Raw data are available in the Supplementary Files.
