# Supplementary material for: Isolation of cancer stem cells by selection for miR-302 expressing cells
Source: PeerJ. 2019 Mar 26;7:e6635. doi: 10.7717/peerj.6635 (PMC6440458; doi:10.7717/peerj.6635)
Supplement: Supplemental Information 1 [file peerj-07-6635-s001.pdf]

Isolation of cancer stem cells by selection for miR-302 expressing cells

Supplementary data:

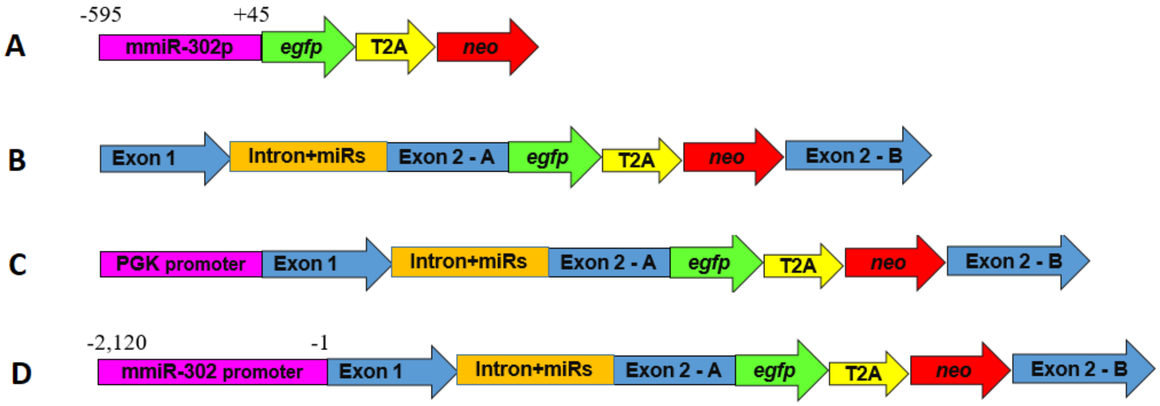

Figure S1: An overview of the constructs designed and used in the project. A) Map of pmmiR302pEGFP-NEO vector, *egfp-neo* is expressed by the core promoter element of murine *miR-302*. B) Map of pmmiR302hostGFP-NEO vector. C) Map of pPGKmm302hostGFP-NEO, *egfp-neo* is expressed by the PGK promoter. D) Map of pmmiR302phostGFP-NEO, *egfp-neo* is expressed by the extended 2.1 kb murine miR-302 promoter.

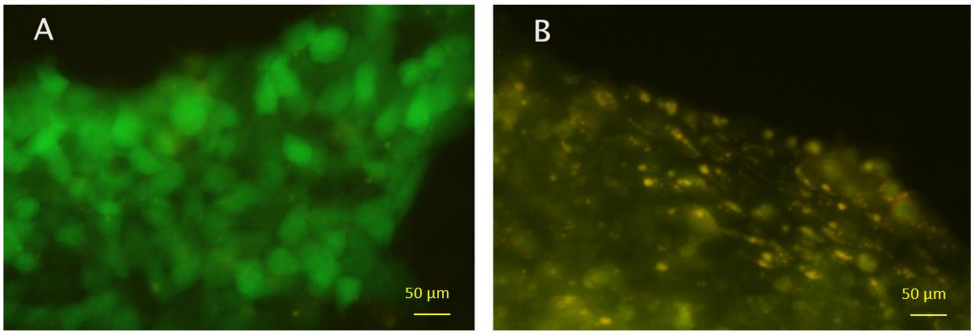

Figure S2: Undifferentiated cells (A), grown for five days on feeder cells with complete ES cell medium containing LIF, showed expression of EGFP under the control of 600 bp *mmiR-302* upstream regulatory sequences. The same clone grown on gelatin with standard fibroblast medium not containing LIF did not show a significant EGFP signal after five days (B).

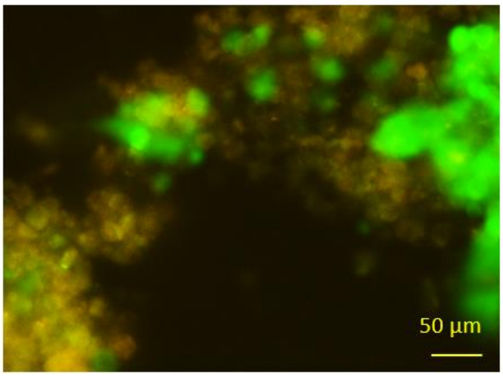

Figure S3: Reappearing EGFP expression after five days of culture under differentiation condition. The distribution of the EGFP signal suggests a reappearing of undifferentiated cells.

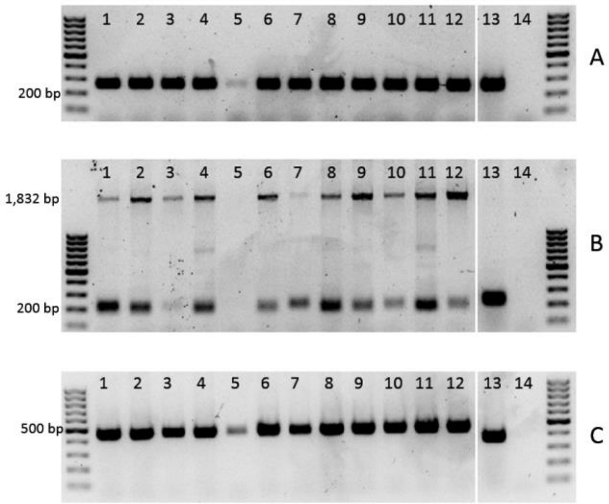

Figure S4: RT-PCR analysis of 12 clones stably transfected with pPGKmmiR302KIGFP-NEO. Lanes 1-12: G418 resistant clone, lane 13: Positive control cDNA from a teratoma, lane 14 is a negative control w/o template cDNA. (A) RT-PCR for *Hprt* as an internal control, the expected band size is 266 bp. (B) RT-PCR using primers in both exons 1 and 2 spanning the *egfp-neo* transgene sequence, the expected band size for the endogenous *mmiR-302* host RNA is 221 bp and 1,832 bp for the transcript contained *egfp-neo* after proper splicing; (C) transgene specific RT-PCR using primers for exon 1 and *egfp*, the expected band size after splicing is 457 bp, but 358 bp for the positive control shown in lane 13.

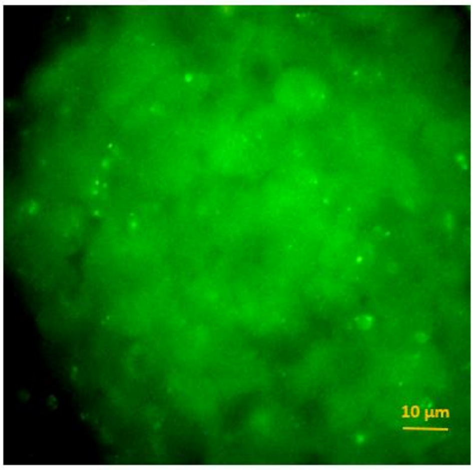

Figure S5: EGFP signal in pPGKmmiR302KIGFP-NEO transfected ES cells.

1500 bp

250 bp

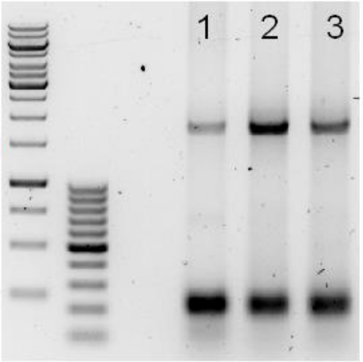

Figure S6: RT-PCR analysis using equal amounts of cDNA from teratoma derived cells under different selection conditions. Lane 1: Primary selected cells (see figure 3); lane 2: Cells stably growing in ES cell medium under the G418 selection; lane 3: Selected cells four days growing in ES cell medium without G418 selection. RT-PCR using primers in both exons 1 and 2 spanning the *egfp-neo* transgene sequence, the expected band size for the endogenous *mmiR-302* host RNA is 221 bp and 1,832 bp for the transgene transcript contained *egfp-neo* after correct splicing.

	Undifferentiated (118 Colonies)			Differentiated (100 Colonies)		
**Relative EGFP signal**	+	+/-	-	+	+/-	-
**Number of colonies (%)**	12 (10.2%)	81 (68.6%)	25 (21.2%)	2 (2%)	33 (33%)	65(65%)

Table S1: From 228 randomly picked primary colonies, 118 colonies grew well enough under ES cell culture condition to be scored. 10.2% of those colonies were completely green, 68.8% showed weak or inhomogeneous EGFP signal and 21.2% were EGFP negative. Under differentiation condition, 100 colonies grew well enough to be scored. Of these only 2% were completely green, 33% showed weak or inhomogeneous EGFP signal and 65% were EGFP negative.

Programs for PCRs and some primer sequences which not mentioned in the text:

All common PCRs were done using this program unless otherwise mentioned:

94 ˚C 5 min, (94 ˚C 1 min, 58 ˚C 45 sec, 72 ˚C 1 min/kb) X 35, 72 ˚C 7 min

mmiR-302 **spliced host RNA RT-PCR:**

94 ˚C 5 min, (94 ˚C 1 min, 56 ˚C 1 min, 72 ˚C 1 min) X 35, 72 ˚C 7 min

Forward primer: 5' AGAACAGGACTCTTTGGGAG

Reverse primer: 5' GCTCCCCAAAAATGTTACTCA

Egfp **and** ***egfp-neo*** **RT-PCR:**

95 ˚C 10 min, (95 ˚C 30 sec, 56 ˚C 30 sec, 72 ˚C 30) X 35, 72 ˚C 7 min

Forward primer for *egfp*: 5' GCGCACCATCTTCTTCAA

Reverse primer for *egfp*: 5' CTTCTCGTTGGGGTCTTT

Forward primer for *egfp-neo*: 5' GGCCGACAAGCAGAAGAA

Reverse primer for *egfp-neo*: 5' CTTGACAAAAAGAACCGGG

Exon1-*egfp* specific RT-PCR:

95 ˚C 10 min, (95 ˚C 45 sec, 53 ˚C 1 min, 72 ˚C 1 min) X 35, 72 ˚C 7 min

Forward primer: 5’ AGAACAGGACTCTTTGGGAG

Reverse primer: 5’ GTCCTTGAAGAAGATGGT

***Hprt* RT-PCR:**

94 ˚C 5 min, (94 ˚C 45 sec, 60 ˚C 45 sec, 72 ˚C 1 min) X 30, 72 ˚C 7 min

Forward primer: 5’ GCAAGCTTGCTGGTGAAAAGGA

Reverse primer: 5’ GCAGAUGGCCACAGGACUAGAACA

Long PCR amplification of the first and second part of the gene:

95 ˚C 10 min, (95 ˚C 1 min, 55 ˚C 1 min, 72 ˚C 2 min) X 35, 72 ˚C 7 min

Primer’s sequences are shown in the text.
