# Supplementary figures and images for: Isolation of cancer stem cells by selection for miR-302 expressing cells
Source: PeerJ. 2019 Mar 26;7:e6635. doi: 10.7717/peerj.6635 (PMC6440458; doi:10.7717/peerj.6635)

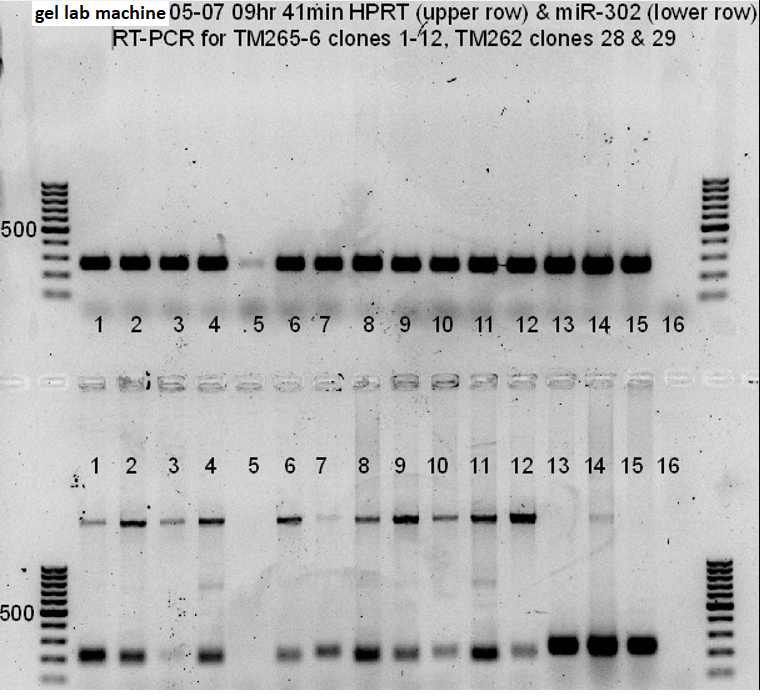

Supplement: Supplemental Information 2 [file peerj-07-6635-s002.zip › RT-PCR analysis of 12 clones stably transfected with pPGKmmiR302KIGFP-NEO (Figure 1 and S4 A-B).png]

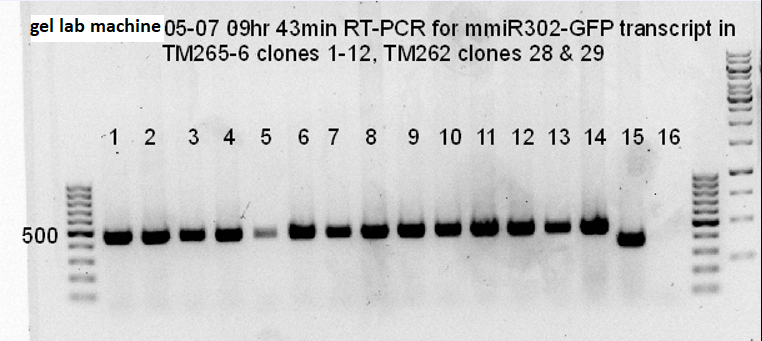

Supplement: Supplemental Information 2 [file peerj-07-6635-s002.zip › RT-PCR analysis of 12 clones stably transfected with pPGKmmiR302KIGFP-NEO (Figure 1 and S4 C).png]

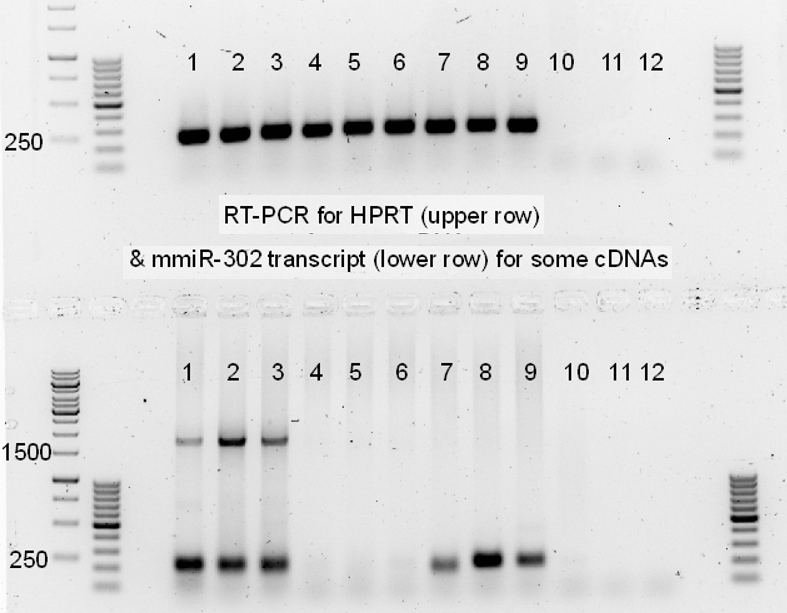

Supplement: Supplemental Information 2 [file peerj-07-6635-s002.zip › RT-PCR analysis of teratoma derived cells (supplement figure S6).png]
